# The RING-Type E3 Ligase BOI Interacts with EXO70E2 and Mediates Its Ubiquitination in *Arabidopsis*

**DOI:** 10.3390/life14091169

**Published:** 2024-09-17

**Authors:** Zhaowu Li, Jianzhong Huang, Yue Hu, Xiaojie Zhou, Xiao Tan, Zhangying Wang, Zhiyong Gao, Xiaoqiu Wu

**Affiliations:** 1 Puai Medical College, Shaoyang University, Shaoyang 422000, China; zhaowuli@whu.edu.cn (Z.L.); 13975960196@163.com (Y.H.); syjjbd@126.com (X.T.); 2 State Key Laboratory of Hybrid Rice, College of Life Sciences, Wuhan University, Wuhan 430072, China; huangjz@whu.edu.cn (J.H.); zywang2017@whu.edu.cn (Z.W.); zygao@whu.edu.cn (Z.G.); 3 College of Food and Chemical Engineering, Shaoyang University, Shaoyang 422000, China; xiaojiezhou@163.com

**Keywords:** *Arabidopsis*, exocyst complex, EXO70E2, BOI

## Abstract

The exocyst is a hetero-octameric complex that exhibits significant functional diversity in regulating biological processes and defense responses. In plants, the EXO70 proteins are important components of the exocyst complex and are involved in membrane trafficking, biotic and abiotic interactions, as well as cell wall formation. A previous study has indicated that a member of the EXO subfamily, EXO70E2, interacts with RIN4 to mediate plant immunity. In this study, we found that EXO70E2 interacts with the RING-type E3 ligase Botrytis susceptible1 interactor (BOI), and the C-terminal domain of BOI is necessary for its interaction with EXO70E2. Moreover, the protein level of EXO70E2 was degraded and ubiquitinated by BOI in vitro. Collectively, our study reveals a mechanism for regulating the stability of EXO70E2 by a RING-type E3 ligase BOI-mediated ubiquitination.

## 1. Introduction

The exocyst complex is an evolutionarily conserved hetero-octameric complex and is required for vesicle trafficking. It is composed of eight subunits, namely Sec3, Sec5, Sec6, Sec8, Sec10, Sec15, EXO70, and EXO84 [1]. In yeasts and humans, the genes that encode each exocyst subunit are single copies. Meanwhile, in land plants, the genes that encode exocyst subunits usually have multiple copies [2,3]. In particular, *EXO70* genes in plants can be divided into three subfamilies (*EXO70.1*, *EXO70.2*, and *EXO70.3*) and nine subgroups (*EXO70A*–*EXO70I*) [4].

The EXO70.1 subfamily contains three members: EXO70A1, EXO70A2, and EXO70A3. *EXO70A1* is constitutively expressed in *Arabidopsis* and it participates in the development of the seed coat, root hairs, the Casparian strip, and tracheary elements (TEs) [5]. An *exo70A1* mutant exhibited dwarf growth, lower fertility, decreased hydraulic transport, as well as reduced PIN transport recycling and cell expansion [6]. EXO70A2 is a closely related isoform of EXO70A1, which participates in pollen maturation. Mutation of *EXO70A2* suppressed pollen tube growth and reduced the pollen germination rate [7]. A protein structure analysis has indicated that only EXO70A3 has transmembrane domains, in contrast to the other EXOs [8].

EXO70.2 is the most evolutionarily dynamic EXO subfamily, which includes six clades, namely EXO70B, C, D, E, F, and H [9]. EXO70B includes two members in *Arabidopsis*, EXO70B1 and EXO70B2. EXO70B1 is involved in the plant immune response and negatively regulates disease resistance. An *exo70B1* mutant exhibited high *PR1* expression and increased contents of SA before pathogen attack [10]. Unlike EXO70B1, EXO70B2 is a positive regulator of disease resistance in *Arabidopsis* and participates in PAMP-triggered immunity [11,12]. *EXO70E2* can recruit exocyst subunits to form exocyst-positive organelles (EXPOs), which are autophagosomes involved in unconventional protein secretion of cytosolic proteins [13]. RIN4 interacts with EXO70E2 at the plasma membrane and alters the localization of EXO70E2 to the cytoplasm. In addition, transient RIN4 expression promotes the transport of EXO70E2 to the extracellular space and enhances the degradation of EXO70E2 [14]. The EXO70.3 includes two clades, namely EXO70G and EXO70I. *EXO70G1* and *EXO70G2* are mainly expressed in the anthers, and mutations in *EXO70G2* cause complete loss of secondary cell wall during TE differentiation [15].

BOI is a RING-type E3 ligase that regulates seed germination, chlorophyll accumulation, and phytohormone signaling. The expression of *BOI* is increased by *B. cinerea* and salt treatment but decreased by gibberellin [16]. The BOI protein has three distinct domains: an N-terminal domain, a conserved central domain that can form a coiled-coil structure, and a C-terminal RING domain. The conserved central domain of BOI can form the coiled-coil structure, and the RING domain, as in most E3 ligases, is required for protein-protein interactions [17]. BOI interacts with the R2R3MYB transcription factor BOS1 and ubiquitinates it in vitro. The BOS1–GUS fusion protein was detected after treatment with MG132, suggesting that BOS1 stability is regulated by the proteasome system [18]. BOI can interact with and ubiquitinate the nucleotide-binding leucine-rich repeat receptor (NLR) protein L5 (AT1G12290) in *N. benthamiana* and *Arabidopsis*, where lysine residue 425 (K425) in the coiled-coil and nucleotide-binding site (CC-NBS) domain of L5 is the main target site for BOI-mediated ubiquitination. In addition, the degradation of L5 (AT1G12290) is dependent on the C-terminal fragment (151–304) of BOI [19]. The BOI-related genes (BRG1, BRG2, and BRG3) are homologs of BOI. The co-expression of BRG1 and BRG3 in *N. benthamiana* decreased the stability of L5 (AT1G12290), suggesting that BOI and its homologs may have functional redundancy in regulating the protein levels of L5 (AT1G12290) [20].

In this study, we found that BOI, as a candidate interactive protein of EXO70E2 from IntAct (https://www.ebi.ac.uk/intact/interaction/EBI-4504968 (accessed on 11 August 2024)), can interact with EXO70E2 both in vitro and in vivo. Moreover, the C-terminal fragment (151–304 amino acid) of BOI is important for its interaction with EXO70E2. Further study indicated that BOI can degrade and ubiquitinate EXO70E2 in vitro. Together, our study indicates that BOI negatively regulates the stability of EXO70E2 via the proteasome system.

## 2. Results

### 2.1. EXO70E2 Interacts with BOI In Vivo and In Vitro

**Figure 1:**
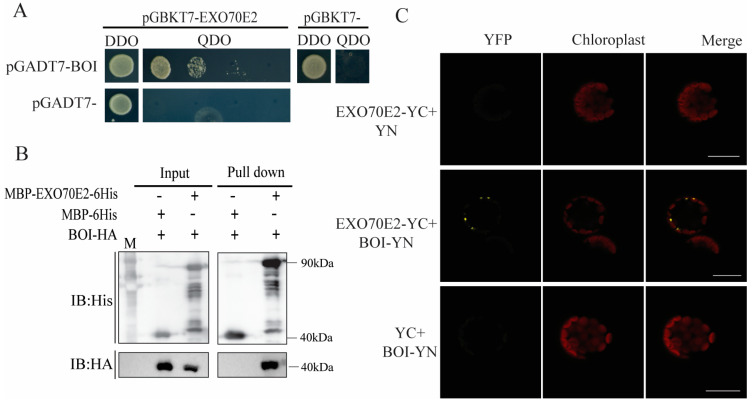
EXO70E2 physically interacts with BOI. (**A**) Interaction of EXO70E2 with BOI in Y2H. EXO70E2 was cloned into pGBKT7, and BOI was cloned into pGADT7, respectively. (**B**) Pull-down assay of EXO70E2 and BOI. Recombinant expressed BOI-HA protein was mixed with MBP-EXO70E2-His or MBP-His Ni-Agarose beads. Precipitated HA-tagged BOI was detected with anti-HA or anti-His antibodies. M: Marker. (**C**) BiFC assay for in vivo interaction between EXO70E and BOI. EXO70E2 fused with C-terminal of YFP and BOI fused with N-terminal of YFP. Combinations of EXO70E2-YFP-YC/YFP-YN, EXO70E2-YFP-YC/BOI-YFP-YN, and YFP-YC/BOI-YFP-YN were co-transformed into protoplasts. Images were captured with a laser scanning confocal microscope. Scale bars = 20 μm. These experiments were repeated three times with similar results.

A previous study has indicated that RIN4 can interact with EXO70E2 and alter its subcellular localization [14]. To better understand the function of EXO70E2, we identified multiple candidate interactive proteins from IntAct, including BOI, MYB73, and WRKY21. BOI is associated with resistance to *Botrytis cinerea* [18], and our studies indicated that overexpression of EXO70E2 decreased resistance to *Botrytis cinerea* (data not published). Based on our results and recent reports, we selected the RING-type protein BOI for validation and further investigation. First, we analyzed the subcellular localization of EXO70E2. The yellow fluorescence of EXO70E2 was observed in the nucleus, while the fluorescence of YFP in the negative control was spread throughout the whole plant cell (Figure S1). These results indicated that EXO70E2 is localized in the nucleus. Previous studies have suggested that BOI is also localized in the nucleus [18], and we demonstrated that BOI is localized in both the nucleus and cytoplasm using the nuclear–cytoplasmic protein fractionation method (Figure S1B). We then analyzed the interaction between EXO70E2 and BOI through a yeast two-hybrid (Y2H) assay. BD-EXO70E2 and AD-BOI were co-transformed into yeast cells. As shown in Figure 1A, the co-transformed BD-EXO70E2 and AD-BOI yeast cells survived on QDO medium. We confirmed the physical interaction of EXO70E2 and BOI via an in vitro pull-down assay. Recombinant MBP-6His, MBP-BOI-HA, and MBP-EXO70E2-6His were produced in *E. coli*. Consistent with the Y2H assay, only MBP-EXO70E2-6His could pull down MBP-BOI-HA, whereas MBP-6His could not (Figure 1B). We further demonstrated the interaction between EXO70E2 and BOI in vivo using a Bimolecular Fluorescence Complementation (BiFC) assay. The results indicated that co-expressed EXO70E2-YC with BOI-YN in protoplasts could reconstitute YFP fluorescence. In contrast, co-expressed EXO70E2-YC with pEarleygate101-YN or pEarleygate101-YC with BOI-YC could not detect YFP fluorescence in protoplasts (Figure 1C). These results demonstrated that EXO70E2 directly interacts with BOI both in vivo and in vitro.

### 2.2. The Protein Level of EXO70E2 Is Negatively Regulated by BOI

**Figure 2:**
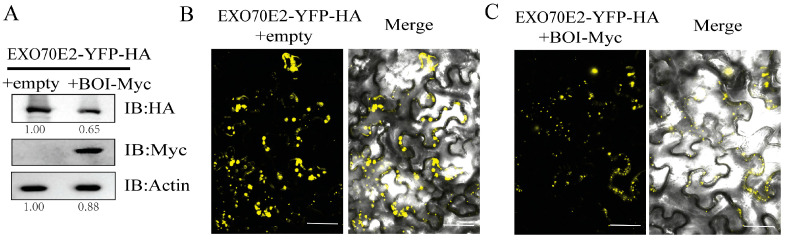
BOI regulates the protein level of EXO70E2. (**A**) EXO70E2-YFP-HA with an empty vector and BOI-Myc were co-expressed in *N. benthamiana*. Immunoblotted proteins were detected using anti-HA antibody or anti-Myc antibody. Actin was used as a loading control. (**B**,**C**) Fluorescence intensity observations. Images were captured with a laser scanning confocal microscope. Scale bars = 50 μm.

BOI is a typical RING-type E3 ligase that mediates the ubiquitination of BOS1 in vitro [18]. To investigate whether BOI affects the protein level of EXO70E2, we co-expressed EXO70E2-YFP-HA with BOI-MYC or an empty vector in the leaves of *Nicotiana benthamiana*. The results indicated that BOI could significantly reduce the protein level of EXO70E2 (Figure 2A). In addition, the YFP fluorescence was significantly decreased and weakened in the co-expressed EXO70E2-YFP-HA and BOI-MYC leaves of *N*. *benthamiana* (Figure 2B,C). Interestingly, the EXO70E2 homolog EXO70E1 could interact with BOI, while the protein level of EXO70E1 was not altered when co-expressed with BOI (Figure S2A,B). Together, these results reveal that BOI can decrease the protein level of EXO70E2, instead of EXO70E1.

BOI has three homologous proteins: BRG1, BRG2, and BRG3. We found that EXO70E2 can interact with BRG2 and BRG3 (Figure S3A). To investigate whether the BRGs are involved in the degradation of EXO70E2, we co–expressed EXO70E2 with BRG1, BRG2, and BRG3 in *N*. *benthamiana*, respectively. Immunoblotting analysis indicated that BRG3, but not BRG2, could decrease the protein level of EXO70E2 (Figure S3B–D). These results suggest that BOI and its homologs may have functional redundancy in regulating the protein level of EXO70E2.

### 2.3. The C-Terminal of BOI Is Crucial for the Degradation of EXO70E

**Figure 3:**
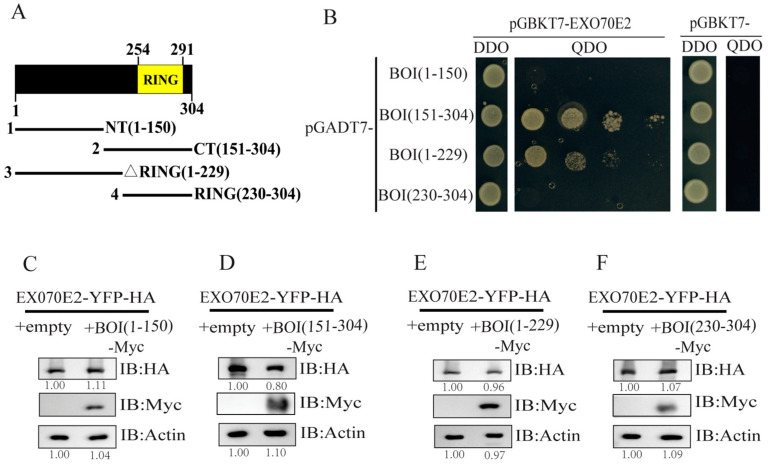
C-terminal of BOI is required to reduce the protein level of EXO70E2. (**A**) Schematic diagram of truncated fragments of BOI. Numbers indicate different amino acid fragments of EXO70E2. (**B**) Interactions of EXO70E2 with truncated fragments of BOI in Y2H. EXO70E2 was cloned into pGBKT7, and truncated fragments of BOI were cloned into pGADT7. (**C**–**F**) EXO70E2 was transiently co-transformed into *N. benthamiana* with empty vector and truncated vectors of BOI. Actin was used as a loading control. These experiments were repeated three times with similar results.

To discern which fragments of BOI are required for its interaction with EXO70E2, we generated a series of BOI truncations: N-terminal (1–150 aa), C-terminal (151–304 aa), RING domain deficient fragment (1–229 aa), and the fragment containing only the RING domain (230–304 aa). Then, these fragments were co-expressed with EXO70E2 in yeast. We found that the C-terminal (151–304 aa) and RING domain deficient fragments (1–229 aa) could interact with EXO70E2, while the N-terminal (1–150 aa) and RING domain fragments (230–304 aa) presented no interaction with EXO70E2 (Figure 3A,B). Moreover, we cloned the truncated fragments of BOI into pEarleygate101 and co-expressed them with EXO70E2 in *N*. *benthamiana*, respectively. The results indicated that the protein level of EXO70E2 was reduced when co-expressed with the C-terminal fragment (151–304 aa) of BOI, while the other fragments of BOI did not affect the protein level of EXO70E2 (Figure 3C–F). These results suggest that the reduction of EXO70E2 protein levels mediated by BOI depends on its C-terminal (151–304 aa).

### 2.4. EXO70E2 Is Ubiquitinated by BOI In Vitro

**Figure 4:**
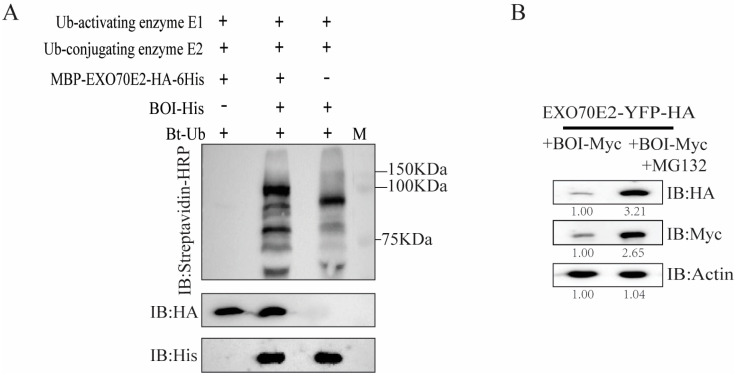
In vitro ubiquitination of EXO70E2 by BOI. (**A**) HA-tagged proteins, His-tagged proteins, and ubiquitinated EXO70E2 proteins were detected using anti-HA antibody, anti-His antibody, and streptavidin-HRP, respectively. M: Marker. (**B**) Proteasome inhibitor MG132 (50 μM) inhibited degradation of EXO70E2 by BOI. Actin was used as a loading control. These xperiments were repeated three times with similar results.

Considering that BOI is an E3 ubiquitin ligase, we hypothesized that EXO70E2 may be modified via BOI-mediated ubiquitination. Therefore, we conducted an in vitro ubiquitination assay to verify whether EXO70E2 is the substrate of BOI. As shown in Figure 4A, the clear protein ubiquitinated form of BOI could be detected when BOI-His was added, indicating that BOI possesses E3 ubiquitin ligase activity. Furthermore, polyubiquitinated bands were detected upon incubation of EXO70E2 with Ub-activating enzyme E1, Ub-conjugating E2, BOI-His, and Bt-Ub. In contrast, no polyubiquitinated bands were detected in reactions without BOI-His (Figure 4A). These results suggest that the degradation and ubiquitination of AtEXO70E2 are mediated by BOI. As the protein level of EXO70E2 is tightly related to the C-terminal of BOI containing the RING domain, we deduced that the degradation of EXO70E2 may be mediated by the 26S proteasome pathway. To confirm this, EXO70E2-YFP-HA and BOI-MYC were co-expressed in *N*. *benthamiana* and then treated with the proteasome inhibitor MG132. As shown in Figure 4B, the protein levels of EXO70E2 and BOI increased after treatment with MG132 (50 μM) for 10 h. These results indicate that MG132 not only inhibits the degradation of EXO70E2 by BOI, but, interestingly, also inhibits the degradation of BOI.

## 3. Discussion

The exocyst complex widely exists in eukaryotes and is highly conserved evolutionarily [21]. The EXO70 family is a crucial component of the exocyst complex and consists of three subfamilies: EXO70.1, EXO70.2, and EXO70.3 [22]. EXO70 family members interact with the small G protein Rho3 and regulate the assembly of SNARE and the exocyst complex [23]. A previous study has indicated that RIN4 interacts with EXO70B1, EXO70E1, EXO70E2, and EXO70F1. Furthermore, RIN4 increases the transport of EXO70E2 to the plasm membrane [14].

In this study, we confirmed the interaction between *EXO70E2* and BOI through Y2H, pull-down, and BiFC assays (Figure 1). Therefore, we hypothesize that BOI may affect the stability of EXO70E2. The obtained results indicate that the protein level of EXO70E2 decreased significantly when EXO70E2 was co-expressed with BOI in *N*. *benthamiana* (Figure 2A–C). Moreover, we found that BOI interacted with EXO70E1 in the Y2H assay but did not influence the protein level of EXO70E1 (Figure S2A,B). These findings suggest that BOI specifically affects the stability of EXO70E2. A previous study has reported that the RING domain is necessary for the function of BOI [19]. Our data indicated that BOI containing only the RING domain is unable to interact with EXO70E2 and, consequently, does not affect the protein level of EXO70E2. However, the C-terminal fragment of BOI that contains the RING domain not only interacted with EXO70E2 but also significantly reduced its protein level (Figure 3). Thus, the C-terminal domain likely plays an important role in the function of BOI.

Protein ubiquitination is considered an important mechanism involved in regulating the stability and variety of proteins [24]. Previous studies have indicated that the U-box ubiquitin ligase PUB18 targets EXO70B1 and regulates the protein level of EXO70B1 through a proteasome-dependent pathway [25]. AvrPtoB is an E3 ligase isolated from *P. syringae* pv. *tomato* DC3000, which interacts with EXO70B2 and mediates the degradation of EXO70B2 via the 26S proteasome system [26]. Considering that BOI is a RING-type E3 ligase and can interact with EXO70E2, we speculated that EXO70E2 might be degraded through the BOI-mediated 26S proteasome pathway. In vitro ubiquitination assays confirmed that EXO70E2 is the substrate of BOI and is directly ubiquitinated by BOI (Figure 4A). In addition, as we found that MG132 could inhibit the degradation of EXO70E2 by BOI, these data indicate that BOI degrades EXO70E2 via the 26S proteasome pathway (Figure 4B). We also found that the degradation of BOI itself was also decreased by MG132 (Figure 4B); these results suggest that BOI not only can ubiquitinate its substrate but also can autoubiquitinate itself. Therefore, it is possible that BOI affects the exocytosis of secretory vesicles through ubiquitination of EXO70E2, and future experiments are required to verify these hypotheses.

Our previous studies showed that RIN4 not only alters the subcellular localization of EXO70E2 but also accelerates the transport of EXO70E2 from vesicles to the extracellular and reduces its protein level [14]. However, the functions of EXO70E2 in vesicles transported to the extracellular space remain unclear. As RIN4, BOI, and EXO70E2 are all associated with plant disease resistance, we hypothesize that BOI and RIN4 may be connected to coordinate their interaction with EXO70E2. Further functional characterization of EXO70E2 is required to clarify the exact relationships between exocytosis and plant growth and development.

## 4. Materials and Methods

### 4.1. Plant Materials and Growth Conditions

The *exo70E2* (SALK_030367) mutant was obtained from the SALK institute. Seeds of the mutant, wild-type, and transgenic *Arabidopsis* were sterilized and grown in a greenhouse at 22 °C, 16 h light/8 h dark, and 70% relative humidity. *N*. *benthamiana* plants were grown in a greenhouse at 24 °C, 12 h light/12 h dark, and 70% relative humidity. The 5-week-old *N. benthamiana* plants were used for subcellular localization and transient expression analyses.

### 4.2. Transient Expression Analysis in N. benthamiana

*Agrobacterium*-mediated transient expression analysis in *N. benthamiana* was performed as previously described [14]. Briefly, expression vectors were transformed into *Agrobacterium* GV3101 and cultured for 10–12 h. The cultured *Agrobacterium* were centrifuged and re–suspended in MES buffer (10 mM MES pH 5.6, 10 mM MgCl_2_, and 150 μM acetosyringone) for 1 h. The diluted suspensions were injected into the leaves of 5-week-old *N. benthamiana* plants. After 48 h of infiltration, the leaves of the plants were used for protein extraction.

### 4.3. Vector Construction

The coding sequences (CDSs) of *EXO70E1*, *EXO70E2*, *BOI*, and truncated *BOI* fragments were cloned into the entry vector pENTR/D-TOPO (Thermo Fisher Scientific, Waltham, MA, USA, #K242020) using a One Step Cloning Kit (Vazyme, Nanjing, China, #C115-02), and the target genes were ligated into the expression vectors (pEarlygate101-YFP, pEarlygate101-N-YFP, pEarlygate101-C-YFP, and pGWB2) with LR Clonase (Thermo Fisher Scientific, Waltham, MA, USA, #11791020).

### 4.4. Immunoblotting Assay

Total proteins were extracted using extraction buffer (20 mM Tris-HCl pH 8.0, 5 mM EDTA, 1% SDS, 10 mM DTT). Protein samples mixed with loading buffer were denatured at 100 °C for 5 min. Equal amounts of total proteins were separated by SDS-PAGE and transferred to PVDF membranes. Immunoblotting was conducted with the following antibodies: anti-HA (Roche, Basel, Switzerland, #11867423001), anti-His (CWBIO, Cambridge, MA, USA, #01249), anti-Myc (Genscript, Piscataway, NJ, USA, #A00704), anti–Histone 3 (AbAbcam, Cambridge, UK, #ab1791), anti-actin (Abbkine, Atlanta, GA, USA, #A01050-2), and anti-ubiquitin (CST, Danvers, MA, USA, #3936) at a 1:10,000 dilution.

### 4.5. Nuclear–Cytoplasmic Protein Fractionation Assay

Nuclear and cytoplasmic proteins were isolated using a previously reported method [27]. Briefly, 1 g of plant leaves was ground in liquid nitrogen and transferred into 4 mL of buffer A. The extracted proteins were filtered through two layers of Miracloth and centrifuged at 1500× *g* at 4 °C for 20 min to separate the crude cytoplasmic and nuclear fractions. Subsequently, the supernatant was centrifuged at 16,000× *g* at 4 °C for 15 min to obtain the cytoplasmic protein. The precipitate was washed with buffer B and buffer C, followed by centrifugation at 16,000× *g* at 4 °C for 20 min. The final precipitate was re-suspended in 200 μL of nuclear lysis buffer.

### 4.6. Yeast Two-Hybrid Assay

The CDS of *EXO70E1* and *EXO70E2* were cloned into pGBKT7, and the CDS of *BOI*, *BRG1*, *BRG2*, and *BRG3* were cloned into pGADT7. The plasmids were then transformed into Gold Yeast Y2H (Clontech, Mountain View, CA, USA, #630498). Yeast cells were grown on SD/(-Trp-Leu) medium for 3 days to select for successful transformation. Single positive colonies were diluted and plated on SD/(-Trp-Leu-His-Ade) medium for 3 days to test the protein-protein interactions between EXO70E2 and BOI.

### 4.7. Pull-Down Assay

The CDS of EXO70E2 and BOI were cloned into pMAL-C2x-6His and pET23b, respectively. The constructed prokaryotic expression vectors were then transformed into *E. coli* BL21 (DE3). Protein expression was induced with 1 mM IPTG when the OD_600_ reached 0.6–1.0, followed by culturing at 16 °C for 20 h. The MBP-BOI-HA protein was incubated with MBP-6His protein or MBP-EXO70E2-6His protein with 500 μL N-NTA agarose beads for 2–3 h at 4 °C. The beads were washed 6–10 times with washing buffer, and the protein-protein interactions between EXO70E2 and BOI were detected via immunoblotting with anti-HA (Roche, #11867423001) antibody at a 1:10,000 dilution.

### 4.8. Bimolecular Fluorescence Complementation (BIFC) Assay

The CDS of *EXO70E2* and *BOI* were cloned into the pENTR/D-TOPO (Thermo Fisher Scientific, Waltham, MA, USA, #K242020) entry vector, then ligated into pEarleygate101-C-YFP and pEarleygate101-N-YFP via the gateway cloning strategy, respectively. Different combinations of expression vectors were co-transformed into *Arabidopsis* protoplasts for 18 h. The YFP signal was observed through a laser scanning confocal microscope (Leica SP8-X, Wetzlar, Germany). A 488 nm wavelength was used to excite the YFP signal and a 525–545 nm wavelength was used to detect the YFP fluorescence.

### 4.9. Ubiquitination Assay

The MBP-EXO70E2-HA-6His and MBP-BOI-6His fusion expression vectors were constructed and transformed into *E. coli* BL21 (DE3), respectively. Protein expression was induced with 0.6 mM IPTG when the OD_600_ reached 0.6–1.0, followed by culturing at 16 °C for 12 h. Recombinant proteins were incubated with 500 μL Ni-NTA agarose beads for 2–3 h at 4 °C. The beads were transferred to a chromatography column and washed with Ni-NTA wash buffer. After the wash buffer had flowed out the column, the beads were re-suspended with Ni–NTA elution buffer. A Bradford Protein Assay Kit (Beyotime, Shanghai, China, #P0012) was used to determine the protein concentration in the elute. The ubiquitination reaction was performed according to the manufacturer’s protocol (Enzo, Farmingdale, NY, USA, #BML-UW9920-0001).

## 5. Conclusions

In this study, we found that EXO70E2 directly interacts with the RING-type E3 ligase BOI both in vivo and in vitro. The C-terminal domain (151–304 amino acids) of BOI is necessary for its interaction with EXO70E2, and BOI can reduce the protein level of EXO70E2 through this C–terminal (151–304 aa) fragment. Additionally, the protein level of EXO70E2 was degraded and ubiquitinated by BOI in vitro. Our study revealed a mechanism for the regulation of EXO70E2 stability mediated by the RING-type E3 ligase BOI through a ubiquitin proteasome pathway. Further experiments are needed to investigate whether BOI affects the exocytosis of secretory vesicles through the ubiquitination of EXO70E2.

## Data Availability

All data are available within this manuscript.

## Supplementary Materials

The following supporting information can be downloaded at: https://www.mdpi.com/article/10.3390/life14091169/s1, Figure S1: Subcellular localization of BOI and EXO70E2. (A) The subcellular localization of BOI fragments in the nuclear-cytoplasmic protein fractionation assay. Total: Total cell proteins. ND: Nuclear-depleted fragments. NE: Nuclear-enriched fragments. (B) EXO70E2 is localized in the nucleus when transiently expressed in *N. benthamiana*. Pictures were captured with a laser scanning confocal microscope. Scale bars = 50 μm; Figure S2: BOI interacts with EXO70E1 but does not alter its protein level. (A) Interaction of EXO70E1 with BOI in Y2H. EXO70E1 was cloned into pGBKT7 and BOI was cloned into pGADT7. (B) EXO70E1-YFP-HA with empty vector or BOI-Myc were co-expressed in *N. benthamiana*. Actin was used as a loading control. These experiments were repeated three times with similar results; Figure S3: The homologs of BOI exhibit functional redundancy in regulating the protein level of EXO70E2. (A) Interaction of EXO70E2 with BRGs in Y2H. EXO70E2 was cloned into pGBKT7 and BRGs were cloned into pGADT7. (B–D) EXO70E2-YFP-HA with empty vector and BRGs-Myc were co-expressed in *N. benthamiana*. Actin was used as a loading control. These experiments were repeated three times with similar results; Table S1: Primers used for PCR amplification and vector construction.
