# Supplementary material for: The RING-Type E3 Ligase BOI Interacts with EXO70E2 and Mediates Its Ubiquitination in Arabidopsis
Source: Life (Basel). 2024 Sep 17;14(9):1169. doi: 10.3390/life14091169 (PMC11432932; doi:10.3390/life14091169)
Supplement: Supplementary file 1 [file life-14-01169-s001.zip › Table S1.pdf]

Table S1. Primers used for PCR amplification and vector construction

Primers used for PCR amplification and vector construction	
primer name	Sequence (5'-3')
pGBDKT7-F	TCATCGGAAGAGAGTAGTAACAAAGGTC
pGBDKT7-R	CCTAAGAGTCACTTTAAAATTTGTATACAC
pGADT7-F	CTATTCGATGATGAAGATACCCCACCAAACCC
pGADT7-R	GTGAACTTGCGGGGTTTTTCAGTATCTACGAT
EXO70E2-F	CAGGCTCCGCGGCCGCCACCATGGCAGAGTTTGATTCCAAGG
EXO70E2-R	AAAGCTGGGTCGGCGCGCCCTCTCTTACGAGAGCTGCGCAG
BOI-F	CAGGCTCCGCGGCCGCCACCATGGCTGTTCAAGCTCATCAC
BOI-R	AAAGCTGGGTCGGCGCGCCCAGAAGACATGTTAACATGCACAC
EXO70E1-F	CAGGCTCCGCGGCCGCCACCATGGGAGAGTTTGAGGTAGATG
EXO70E1-R	AAAGCTGGGTCGGCGCGCCCTCTTCTATAAGAATTGTTCAAGG
BRG1-F	CAGGCTCCGCGGCCGCCACCATGGCTGTTGAAGCAAGACACATG
BRG1-R	AAAGCTGGGTCGGCGCGCCCTGATGACATGTTAACATGTACAC
BRG2-F	CAGGCTCCGCGGCCGCCACCATGGCCGTCGATGCTCACCATCTC
BRG2-R	AAAGCTGGGTCGGCGCGCCCAGATGACATGTTGACATGAACGC
BRG3-F	CAGGCTCCGCGGCCGCCACCATGGCCGTTGAAGCTCACCATCTAAATC
BRG3-R	AAAGCTGGGTCGGCGCGCCCAGAGGAAAGATTAACATGTAGACTAGC
BOI (1-150)-F	CAGGCTCCGCGGCCGCCACCATGGCTGTTCAAGCTCATCAC
BOI (1-150)-R	AAAGCTGGGTCGGCGCGCCCCGCTAACATCCGCGTTTGC
BOI (151-304)-F	CAGGCTCCGCGGCCGCCACCATGTCTGCGGTTCAAAACGTTATAGC
BOI (151-304)-R	AAAGCTGGGTCGGCGCGCCCAGAAGACATGTTAACATGCACAC
BOI (1-229)-F	CAGGCTCCGCGGCCGCCACCATGGCTGTTCAAGCTCATCAC
BOI (1-229)-R	AAAGCTGGGTCGGCGCGCCCGTCTTCTACAACGGCTGAAGCG
BOI (230-304)-F	CAGGCTCCGCGGCCGCCACCATGGATGCGGAATCGAGTTGTGG
BOI (230-304)-R	AAAGCTGGGTCGGCGCGCCCAGAAGACATGTTAACATGCACAC
