# Supplementary figures and images for: The RING-Type E3 Ligase BOI Interacts with EXO70E2 and Mediates Its Ubiquitination in Arabidopsis
Source: Life (Basel). 2024 Sep 17;14(9):1169. doi: 10.3390/life14091169 (PMC11432932; doi:10.3390/life14091169)

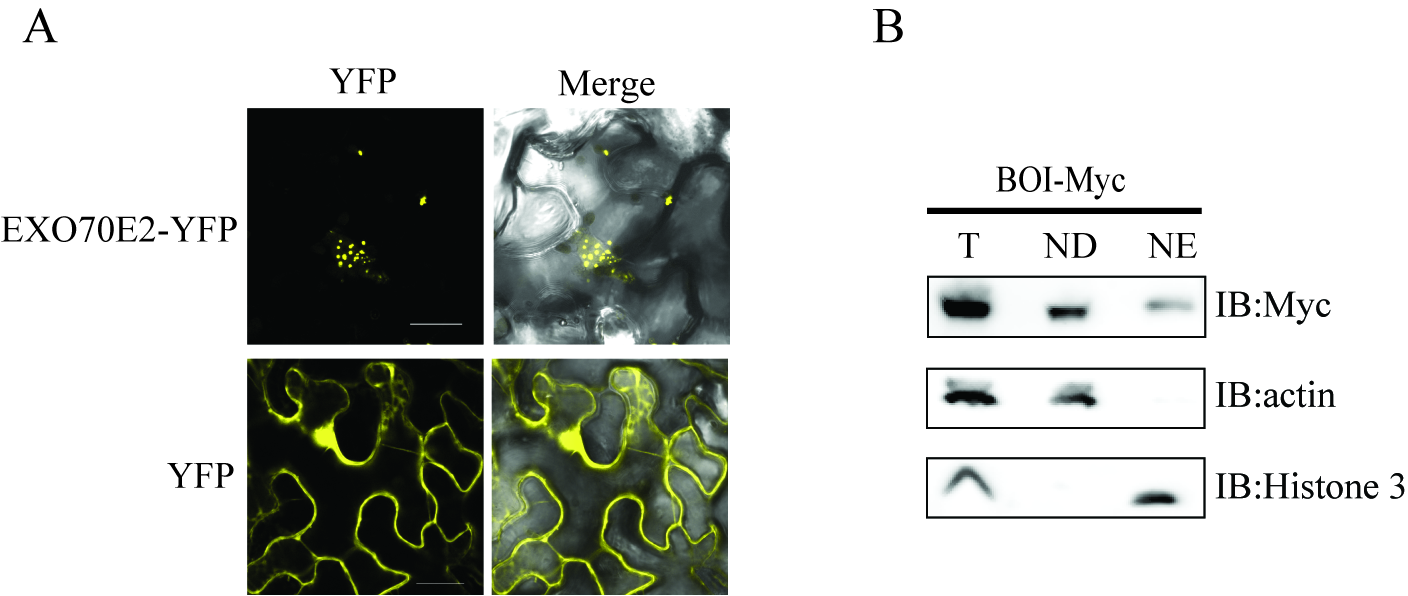

Supplement: Supplementary file 1 [file life-14-01169-s001.zip › Fig S1.tif]

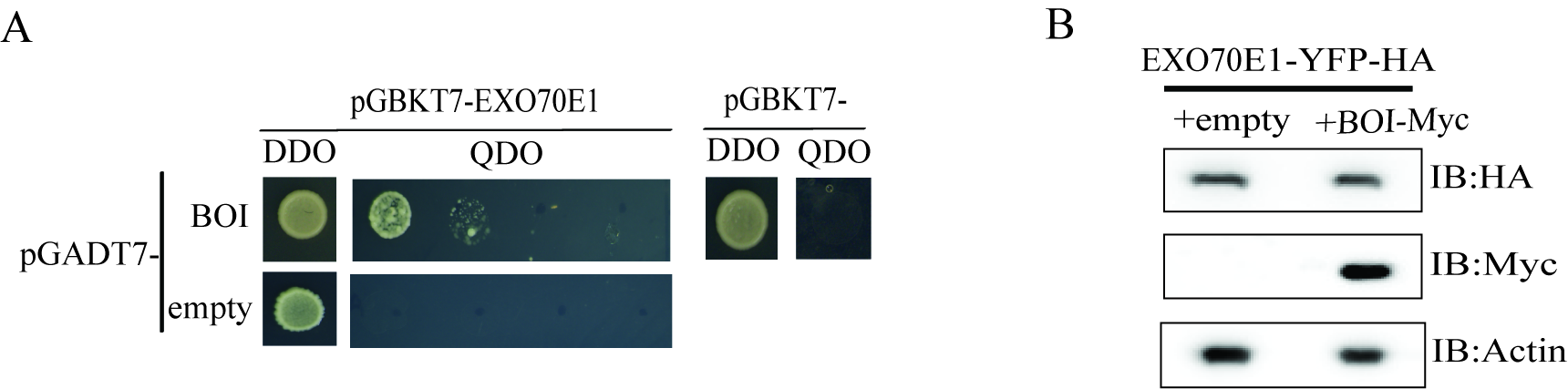

Supplement: Supplementary file 1 [file life-14-01169-s001.zip › Fig S2.tif]

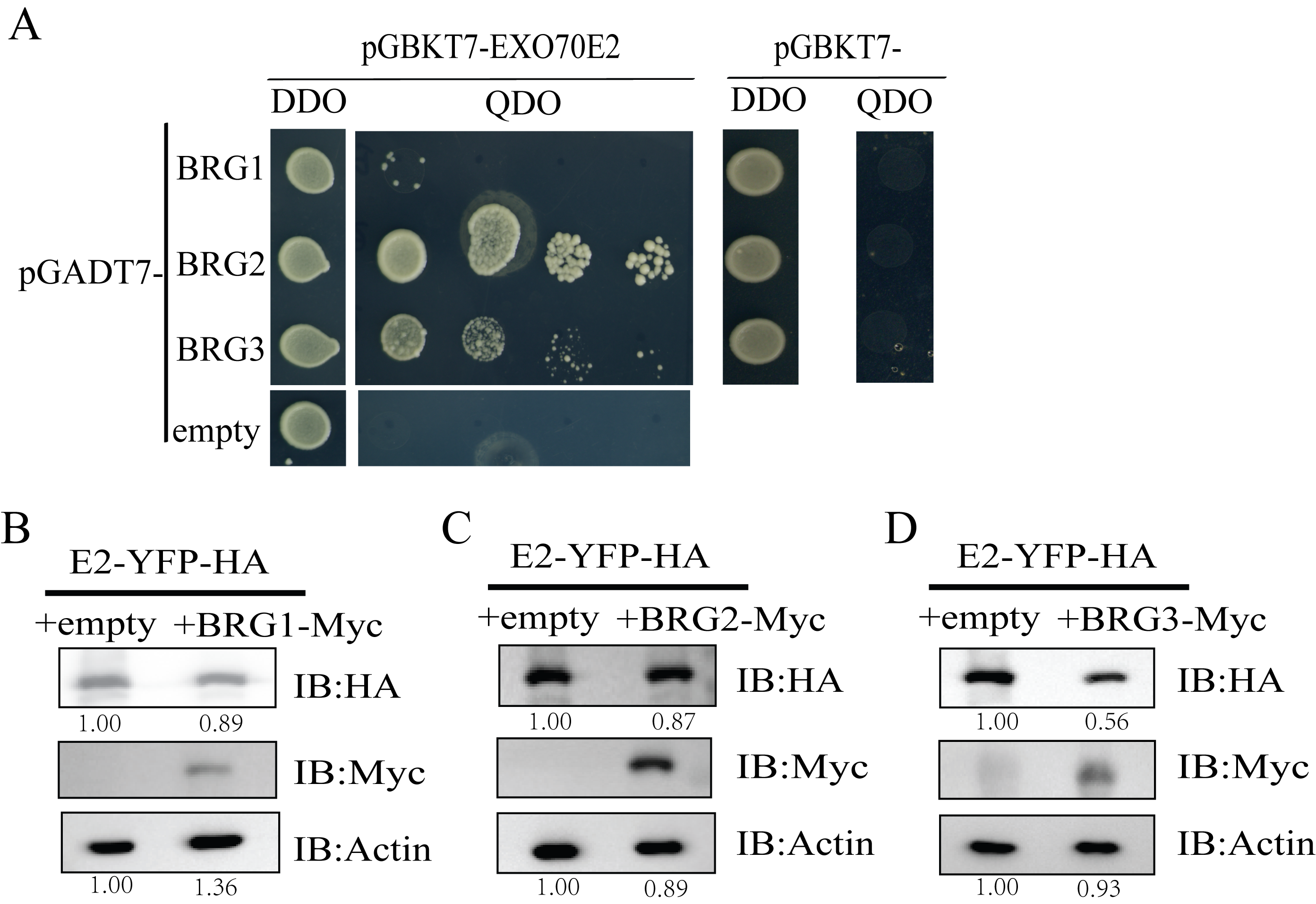

Supplement: Supplementary file 1 [file life-14-01169-s001.zip › Fig S3.tif]
